# Integrated Methylome and Transcriptome Analyses Reveal Methylation-Associated Cadmium Stress Responses in *Sophora tonkinensis*

**DOI:** 10.3390/plants15121861

**Published:** 2026-06-16

**Authors:** Fan Wei, Shuangshuang Qin, Yang Lin, Linxuan Li, Guili Wei, Danfeng Tang, Meiqiong Tang, Ying Liang

**Affiliations:** Guangxi Key Laboratory of Medicinal Resources Protection and Genetic Improvement, Guangxi Engineering Research Center of TCM Resource Intelligent Creation, National Center for TCM Inheritance and Innovation, Guangxi Botanical Garden of Medicinal Plants, Nanning 530023, China

**Keywords:** cadmium stress, DNA methylation, WGBS, RNA-seq, methylation-associated responses, *Sophora tonkinensis*

## Abstract

Cadmium (Cd) is a highly toxic heavy metal that impairs plant growth, metabolism, and the accumulation of bioactive compounds. To investigate methylation-associated Cd responses in the medicinal plant *Sophora tonkinensis*, we integrated whole-genome bisulfite sequencing (WGBS) and transcriptome sequencing under three Cd treatments (T0, T2, and T4). Cd stress induced extensive transcriptional reprogramming and widespread DNA methylation changes, with CHH methylcytosines accounting for the largest proportion of methylated sites, whereas CG sites showed the highest average methylation level. Differentially methylated regions (DMRs) were predominantly detected in the CHH context and were frequently located in promoter and flanking regions. Integrated analysis identified 6547 and 6204 differentially methylated genes in T2 vs. T0 and T4 vs. T0, respectively, and 420 and 612 genes, respectively, showing concurrent changes in DNA methylation and transcript abundance. Genes with hypermethylation and reduced expression were more frequent than hypomethylated/upregulated genes and were mainly associated with photosynthesis, carbon fixation, fatty acid metabolism, sulfur-related metabolism, and secondary metabolic pathways potentially related to medicinal quality. Among the hypomethylated/upregulated genes, the hormone-related candidate gene *StGH3.1* was selected for functional validation, and heterologous overexpression of *StGH3.1* enhanced Cd tolerance in transgenic *Nicotiana benthamiana*. These results indicate that Cd stress is accompanied by coordinated methylome and transcriptome remodeling in *S. tonkinensis* and provide methylation-associated candidate genes for further investigation of Cd-responsive adaptation.

## 1. Introduction

Cadmium (Cd) contamination has become an increasingly important constraint in plant production because it suppresses growth, disturbs metabolism, and impairs overall physiological performance. In medicinal plants, this issue is particularly critical because Cd stress may affect not only biomass accumulation but also the biosynthesis, transport, and stability of pharmacologically active constituents [1,2,3]. In *Sophora tonkinensis*, Cd exposure has already been shown to impair growth and alter physiological and metabolic traits, indicating that this medicinal species is sensitive to Cd toxicity at both the agronomic and quality-related levels [4].

Plants respond to Cd toxicity through coordinated defense systems rather than through a single protective mechanism. These responses include restriction of metal uptake and translocation, chelation and vacuolar sequestration, antioxidant activation, transporter-mediated detoxification, and transcriptional reprogramming [5,6,7]. In addition, nutrient-element status, silicon supplementation, and transcription factor networks can strongly influence the extent of Cd injury and tolerance, further indicating that plant adaptation to Cd stress is an actively regulated process [8,9,10]. Although these physiological and molecular responses have been widely investigated, the upstream epigenetic basis linking stress perception to transcriptional and metabolic reprogramming remains insufficiently understood, especially in medicinal plants [2,11].

Among the epigenetic mechanisms involved in abiotic-stress adaptation, DNA methylation has attracted particular attention because it can influence chromatin state and gene activity without altering DNA sequence [11,12,13,14]. In plants, cytosine methylation occurs in CG, CHG, and CHH contexts, and increasing evidence suggests that methylation dynamics are closely associated with responses to heavy metal stress, redox homeostasis, and broader environmental adaptation [15,16,17,18]. In *S. tonkinensis*, genome-wide analysis of methylation-related genes has already shown that members of the C5-MTase and DNA demethylase families respond to cadmium and drought stress, suggesting that DNA methylation machinery itself is stress responsive in this species [19]. Our previous transcriptome and small-RNA study under drought also suggested that *S. tonkinensis* possesses complex stress-responsive regulatory networks at the RNA level [20].

Recent integrated methylome–transcriptome studies in multiple plant systems have shown that stress-induced methylation remodeling can be associated with changes in gene expression related to photosynthesis, carbon metabolism, signaling, and abiotic-stress defense [21,22,23,24,25,26]. At the same time, transcriptomic and multi-omics studies in medicinal and non-medicinal plants under Cd stress have consistently highlighted the importance of detoxification, antioxidant regulation, transporter activity, lipid metabolism, hormone signaling, and secondary metabolism in stress adaptation [27,28,29,30]. Together, these studies suggest that the key challenge is no longer simply to catalog Cd-responsive genes, but to determine how genome-wide epigenetic remodeling is linked to transcriptional outputs and quality-related metabolism in medicinal plants.

This question is particularly relevant for *S. tonkinensis*, a medicinal species in which Cd stress may affect both plant performance and medicinal value. Our previous physiological and metabolomic study showed that Cd exposure induced substantial changes in growth-related traits and metabolite accumulation [4], while our subsequent gene-family analysis indicated that DNA methylation-related regulators are themselves responsive to Cd and drought [19]. However, despite these advances, genome-scale evidence linking DNA methylation changes with transcriptomic reprogramming under Cd stress is still lacking in *S. tonkinensis*.

To address this gap, we combined whole-genome bisulfite sequencing (WGBS) and transcriptome sequencing to characterize the methylation and transcriptional landscapes of *S. tonkinensis* under different Cd treatments. By integrating methylome and transcriptome datasets, we aimed to identify Cd-responsive methylation patterns, determine how DNA methylation changes are associated with transcript abundance, and reveal candidate pathways and genes potentially involved in stress adaptation and medicinally relevant metabolism. This study provides a genome-wide framework for understanding the methylation-associated Cd responses in *S. tonkinensis*.

## 2. Results

### 2.1. Cd Stress Induced Extensive Transcriptional Reprogramming in S. tonkinensis

**Figure 1:**
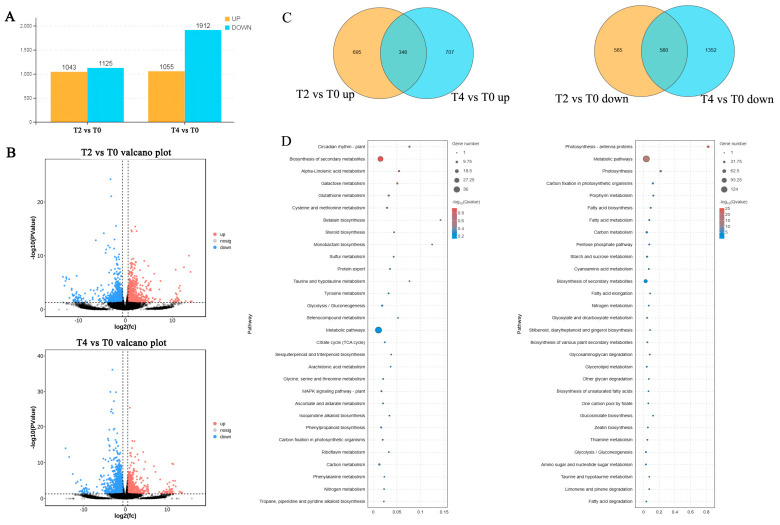
Transcriptome responses of *S. tonkinensis* under Cd stress. (**A**) Numbers of upregulated and downregulated genes identified in the T2 vs. T0 and T4 vs. T0 comparisons. (**B**) Volcano plots of differentially expressed genes in the T2 vs. T0 and T4 vs. T0 comparisons. (**C**) Venn diagrams showing commonly upregulated and commonly downregulated genes shared between the two comparisons. (**D**) KEGG enrichment analysis of the common upregulated and downregulated genes, respectively.

Transcriptome profiling revealed that Cd exposure triggered substantial changes in gene expression in *S. tonkinensis*. In the T2 vs. T0 comparison, 1043 genes were upregulated and 1125 genes were downregulated, whereas in the T4 vs. T0 comparison, 1055 genes were upregulated and 1912 genes were downregulated (Figure 1A,B). The markedly higher number of downregulated genes in T4 vs. T0 suggests that more severe Cd stress exerted a stronger inhibitory effect on the transcriptome. The detailed RNA-seq quality-control and alignment statistics are listed in Supplementary Table S1.

Venn analysis further showed that 348 upregulated genes and 560 downregulated genes were shared between the T2 vs. T0 and T4 vs. T0 comparisons (Figure 1C), indicating the presence of both a conserved Cd-responsive transcriptional program and treatment-intensity-dependent regulatory changes. KEGG enrichment analysis showed that the 348 commonly upregulated genes were mainly involved in circadian rhythm–plant, biosynthesis of secondary metabolites, alpha-linolenic acid metabolism, galactose metabolism, glutathione metabolism, and cysteine and methionine metabolism. In contrast, the 560 commonly downregulated genes were primarily enriched in photosynthesis, carbon fixation in photosynthetic organisms, porphyrin metabolism, fatty acid biosynthesis, fatty acid metabolism, carbon metabolism, the pentose phosphate pathway, starch and sucrose metabolism, and cyanoamino acid metabolism (Figure 1D).

### 2.2. Global DNA Methylation Was Remodeled Under Cd Stress, Whereas Its Overall Distribution Across Gene Regions Remained Largely Conserved

**Figure 2:**
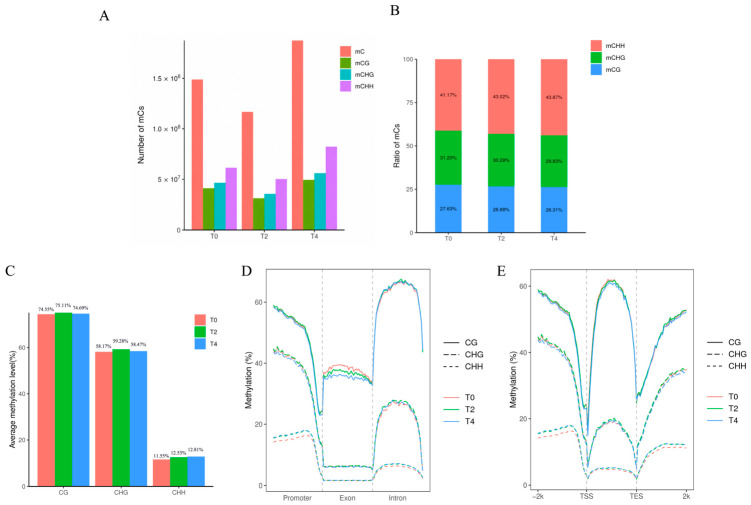
Global DNA methylation patterns of *S. tonkinensis* under Cd stress. (**A**,**B**) Number and relative proportion of methylated cytosine sites in CG, CHG, and CHH contexts in the T0, T2, and T4 groups. (**C**) Average methylation levels within each cytosine context in the T0, T2, and T4 groups. (**D**) Methylation profiles across promoter, exon, and intron regions. (**E**) Methylation profiles across upstream, gene body, and downstream regions.

The detailed WGBS sequencing and mapping statistics are listed in Supplementary Table S2. WGBS analysis revealed clear alterations in genome-wide DNA methylation patterns under Cd stress. Across all treatments, CHH methylcytosines accounted for the largest proportion of methylated cytosine sites, increasing from 41.17% in T0 to 43.02% in T2 and 43.87% in T4, whereas CG sites represented the smallest proportion, decreasing from 27.63% in T0 to 26.68% in T2 and 26.31% in T4 (Figure 2A,B). These values reflect the relative composition of methylated cytosines among the CG, CHG, and CHH contexts. We further calculated the average methylation level within each cytosine context to evaluate context-specific methylation intensity. Although CG sites accounted for the smallest proportion of methylated cytosines, they exhibited the highest average methylation level, followed by CHG and CHH sites. Specifically, the average methylation levels of CG sites were 74.55%, 75.11%, and 74.69% in T0, T2, and T4, respectively, while those of CHG sites were 58.17%, 59.28%, and 58.47%, and those of CHH sites were 11.55%, 12.53%, and 12.81%, respectively (Figure 2C). These results indicate that Cd treatment induced methylation changes in all three sequence contexts, with the most pronounced increase occurring in the CHH context.

To further characterize the overall methylation patterns across gene-associated regions, we examined methylation levels in promoter, exon, and intron regions, as well as across upstream, gene body, and downstream regions (Figure 2D,E). In all three treatments, CG methylation remained consistently higher than CHG and CHH methylation across most genomic regions. In the promoter–exon–intron analysis, exons generally showed lower methylation levels than promoters and introns. In the upstream–gene body–downstream analysis, methylation levels decreased markedly near the transcription start site (TSS) and transcription end site (TES), whereas relatively higher methylation levels were detected in the flanking regions. Although the overall distribution patterns were largely similar among T0, T2, and T4, differences in methylation intensity were still observed among sequence contexts and genomic regions. Taken together, these findings indicate that Cd stress in *S. tonkinensis* primarily affected DNA methylation levels, particularly in the CHH context, rather than fundamentally altering the overall distribution pattern of methylation across gene regions.

### 2.3. Cd Stress Induced Extensive CHH-Dominant DMRs Predominantly Located in Promoter and Flanking Regions

**Figure 3:**
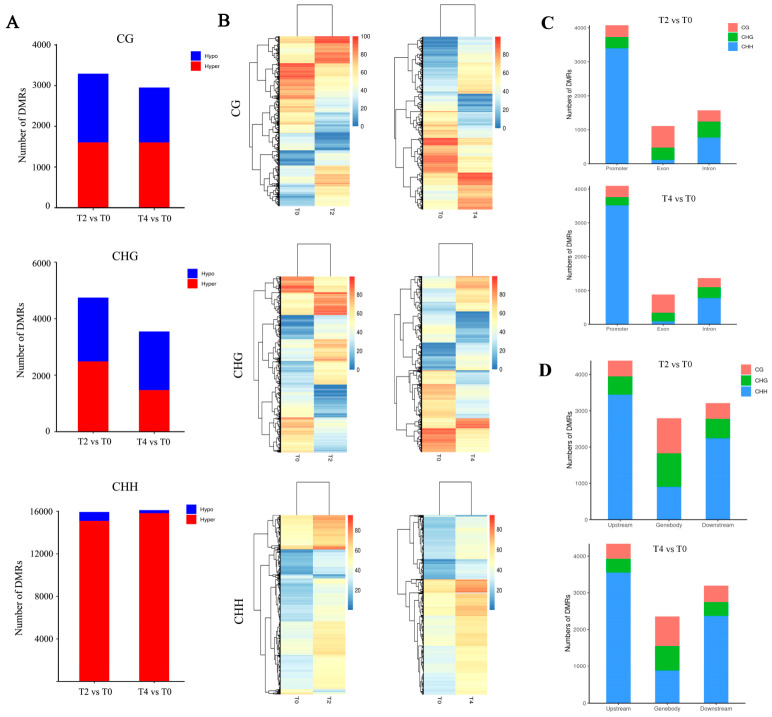
Cd-responsive differentially methylated regions (DMRs) in *S. tonkinensis*. (**A**) Numbers of hypermethylated and hypomethylated DMRs in CG, CHG, and CHH contexts in the T2 vs. T0 and T4 vs. T0 comparisons. (**B**) Heatmap showing methylation differentiation between control and Cd-treated samples. (**C**) Distribution of DMRs across promoter, exon, and intron regions in the T0 vs. T2 and T0 vs. T4 comparisons, respectively. (**D**) Distribution of DMRs across upstream, gene body, and downstream regions in the T0 vs. T2 and T0 vs. T4 comparisons, respectively.

Large numbers of differentially methylated regions (DMRs) were identified under Cd stress, with approximately 25,000 DMRs detected in T2 vs. T0 and approximately 24,000 in T4 vs. T0. Among the three sequence contexts, CHH DMRs represented the largest fraction of methylation changes, and most CHH DMRs were hypermethylated, whereas CG and CHG DMRs displayed more balanced proportions of hyper- and hypomethylation (Figure 3A). Heatmap analysis also revealed clear methylation differentiation between control and Cd-treated samples (Figure 3B). Annotation of DMRs to genomic features showed that, among the annotated genomic regions, promoter regions contained the largest number of DMRs in both comparisons (Figure 3C). In addition, introns generally harbored more DMRs than exons, and upstream and downstream regions contained substantially more DMRs than gene bodies, particularly in the CHH context (Figure 3D). Together, these findings indicate that Cd stress induced extensive CHH-dominant methylation variation, with DMRs more frequently detected in promoter and flanking regions, which may be associated with transcriptional regulation during the Cd stress response.

### 2.4. Integrated Analysis Identified Methylation-Associated Genes in Cd Stress-Responsive and Secondary-Metabolism-Related Pathways

**Figure 4:**
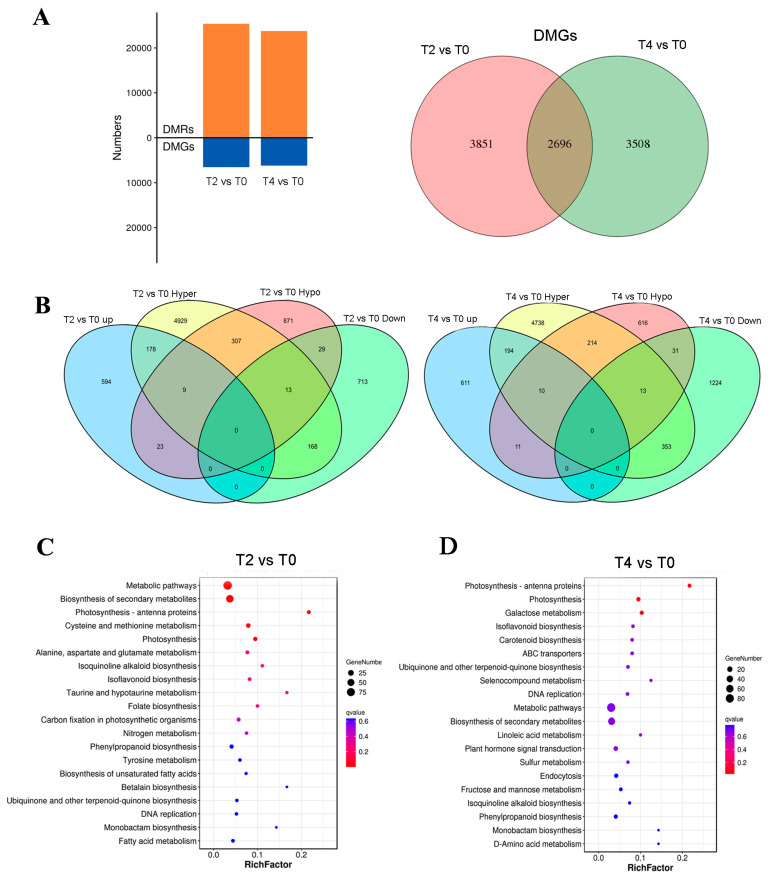
Integrated analysis of DNA methylation and transcript abundance under Cd stress. (**A**) Numbers of differentially methylated genes (DMGs) identified in T2 vs. T0 and T4 vs. T0 and the DMGs shared between the two comparisons. (**B**) Venn diagram analysis of DMGs and DEGs. (**C**,**D**) KEGG enrichment analyses of genes showing simultaneous changes in methylation and transcript expression in the T2 vs. T0 and T4 vs. T0 comparisons, respectively.

A total of 6547 differentially methylated genes (DMGs) were identified in T2 vs. T0 and 6204 in T4 vs. T0, of which 2696 were shared by both comparisons. This substantial overlap indicates that a common set of genes underwent methylation remodeling under Cd stress, while others responded in a treatment-specific manner (Figure 4A). To investigate the relationship between DNA methylation and gene expression, WGBS and transcriptome data were integrated separately for each comparison. A total of 420 genes in T2 vs. T0 and 612 genes in T4 vs. T0 exhibited simultaneous changes in both methylation level and transcript abundance (Figure 4B).

Functional enrichment analysis of genes showing concurrent changes in DNA methylation and transcript abundance identified several pathways closely associated with the Cd stress response. In the T2 vs. T0 comparison, these genes were mainly enriched in metabolic pathways, biosynthesis of secondary metabolites, photosynthesis-antenna proteins, photosynthesis, cysteine and methionine metabolism, carbon fixation in photosynthetic organisms, isoquinoline alkaloid biosynthesis, and fatty acid metabolism (Figure 4C). These pathways suggest that moderate Cd stress affected not only photosynthetic capacity and primary metabolism, but also sulfur-containing amino acid metabolism and isoquinoline alkaloid biosynthesis. In the T4 vs. T0 comparison, these genes were primarily enriched in photosynthesis-antenna proteins, photosynthesis, galactose metabolism, carotenoid biosynthesis, ABC transporters, biosynthesis of secondary metabolites, plant hormone signal transduction, and sulfur metabolism (Figure 4D). The enrichment of ABC transporters, hormone signaling, and sulfur metabolism indicates that stronger Cd stress may trigger more pronounced detoxification, transport, and stress signaling responses. Notably, the repeated detection of biosynthesis of secondary metabolites and isoquinoline alkaloid biosynthesis in the integrated analysis suggests that Cd-responsive methylation-associated genes may be linked not only to general stress-adaptation, but also to secondary metabolic processes potentially related to the medicinal quality of *S. tonkinensis*.

To further examine the relationship between DNA methylation and gene expression at the regional level, we compared transcript abundance among genes grouped by methylation levels in promoter and gene body regions under T0, T2, and T4 (Figures S1 and S2). Distinct context-dependent patterns were observed in both regions. In promoters, CHG and CHH methylation tended to be associated with reduced expression, whereas the relationship for CG methylation was less uniform. In gene bodies, CG methylation was generally associated with relatively higher expression at intermediate-to-high methylation levels, while CHG and CHH methylation showed overall negative associations with transcript abundance. This analysis was intended to describe population-level associations between regional methylation status and transcript abundance, rather than to identify individual Cd-responsive DMR-DEG candidate genes.

### 2.5. Common Hypermethylated/Downregulated Genes in Both Comparisons Were Mainly Enriched in Photosynthesis, Lipid Metabolism, and Secondary Metabolism

**Figure 5:**
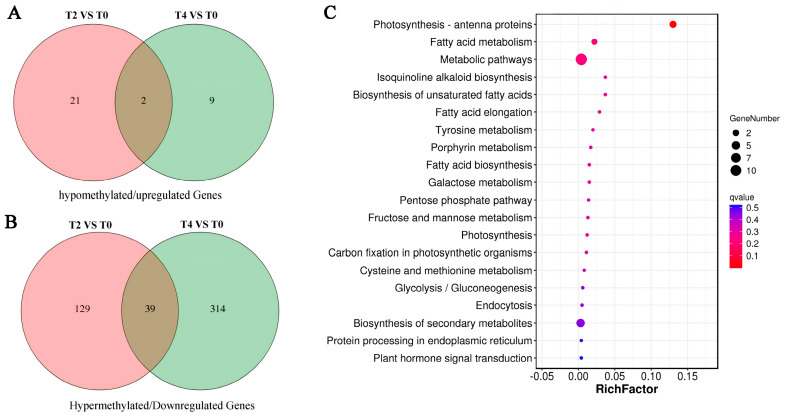
Comparative analysis of genes showing inverse relationships between DNA methylation and gene expression in the T2 vs. T0 and T4 vs. T0 comparisons. (**A**) Venn diagram showing shared hypomethylated/upregulated genes. (**B**) Venn diagram showing shared hypermethylated/downregulated genes. (**C**) KEGG enrichment analysis of the shared hypermethylated/downregulated genes.

To further identify candidate genes in which DNA methylation changes may be associated with transcriptional responsiveness under Cd stress, we focused on genes showing inverse relationships between DNA methylation and gene expression, namely hypomethylated/upregulated and hypermethylated/downregulated genes. In T2 vs. T0, 23 genes were hypomethylated and upregulated, whereas 168 genes were hypermethylated and downregulated. In T4 vs. T0, 11 genes belonged to the hypomethylated/upregulated group, whereas 353 genes belonged to the hypermethylated/downregulated group (Figure 5A,B). The predominance of hypermethylated/downregulated genes, particularly under the higher Cd treatment, suggests that genes showing concurrent increases in methylation and decreases in transcript abundance became more frequent as Cd stress intensified.

To further identify common methylation-associated patterns under Cd stress, we compared these two gene groups between T2 vs. T0 and T4 vs. T0. Only two genes were shared between the hypomethylated/upregulated groups of the two comparisons. One was annotated as a hypothetical protein, whereas *StGH3.1* was mapped to the plant hormone signal transduction pathway. By contrast, 39 genes were shared between the hypermethylated/downregulated groups of T2 vs. T0 and T4 vs. T0 (Figure 5C). KEGG enrichment analysis showed that these 39 common hypermethylated/downregulated genes were mainly involved in photosynthesis-antenna proteins, fatty acid metabolism, metabolic pathways, isoquinoline alkaloid biosynthesis, biosynthesis of unsaturated fatty acids, photosynthesis, and carbon fixation in photosynthetic organisms. Additional enriched pathways included cysteine and methionine metabolism, galactose metabolism, fatty acid biosynthesis, glycolysis/gluconeogenesis, biosynthesis of secondary metabolites, and plant hormone signal transduction. These results indicate that the common genes showing increased methylation together with reduced expression under Cd stress were mainly associated with photosynthetic activity, primary and lipid metabolism, sulfur-related metabolism, and secondary metabolic pathways potentially related to medicinal quality in *S. tonkinensis*, including isoquinoline alkaloid biosynthesis.

### 2.6. Heterologous Overexpression of StGH3.1 Enhanced Cadmium Tolerance in Transgenic Nicotiana benthamiana

**Figure 6:**
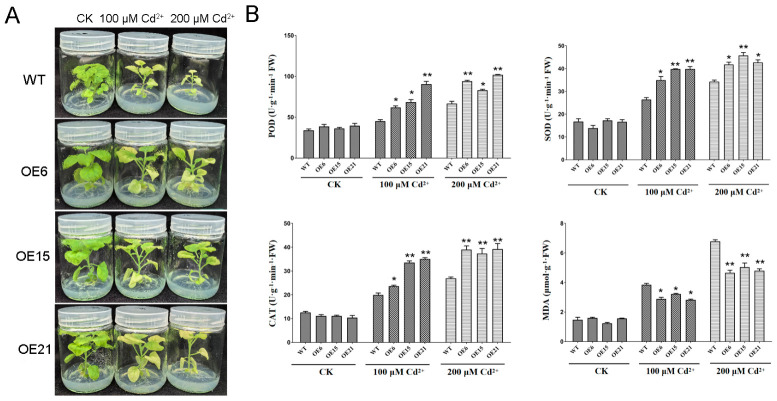
Functional validation of *StGH3.1* in transgenic *Nicotiana benthamiana* under Cd stress. (**A**) Growth phenotypes of wild-type *N. benthamiana* and three *StGH3.1*-overexpressing lines (OE6, OE15, and OE21) under control, 100 μM CdCl_2_, and 200 μM CdCl_2_ treatments for 30 d. (**B**) POD, SOD, and CAT activities and MDA content in leaves of wild-type and *StGH3.1*-overexpressing plants under Cd stress. Data are presented as mean ± SD. Asterisks indicate significant differences between the transgenic lines and the wild type under the same treatment (* *p* < 0.05, ** *p* < 0.01).

To further evaluate whether methylation-associated candidate genes contribute to Cd tolerance, *StGH3.1* was selected for heterologous validation. *StGH3.1* was one of the two genes shared between the hypomethylated/upregulated groups in both Cd comparisons, and it was mapped to the plant hormone signal transduction pathway, consistent with its annotation as a GH3 auxin-responsive gene. Locus-level inspection of the WGBS data showed that the DMR associated with *StGH3.1* was located in the upstream region of the gene and displayed a decreasing methylation trend from T0 to T2 to T4 (Figure S3). Because of its clear functional annotation and potential involvement in hormone-related stress regulation, we examined whether heterologous overexpression of *StGH3.1* could enhance Cd tolerance in transgenic *Nicotiana benthamiana*. qRT-PCR analysis confirmed that OE6, OE15, and OE21 exhibited relatively high expression levels of *StGH3.1* and were therefore selected for subsequent analyses (Figure S4). After 30 d of Cd exposure, clear phenotypic differences were observed between the wild type and the transgenic lines. Under both 100 and 200 μM CdCl_2_ treatments, the *StGH3.1*-overexpressing lines OE6, OE15, and OE21 generally showed better growth status than the wild type, indicating that heterologous expression of *StGH3.1* enhanced plant tolerance to Cd stress in *N. benthamiana* (Figure 6A).

To further characterize the physiological responses of the independent overexpression lines, we measured POD, SOD, and CAT activities and MDA content separately in WT, OE6, OE15, and OE21. Under Cd stress, all three overexpression lines generally showed higher POD, SOD, and CAT activities and lower MDA content than the wild type, although the magnitude of these responses varied among lines and treatments (Figure 6B). These results indicate that *StGH3.1* overexpression enhanced Cd tolerance in transgenic *N. benthamiana*, at least in part, by improving antioxidant capacity and reducing oxidative damage.

## 3. Discussion

### 3.1. Cd Stress Induces Coordinated Transcriptomic and Methylomic Changes in S. tonkinensis

The present study shows that Cd stress in *S. tonkinensis* induces substantial changes in both gene expression and DNA methylation (Figure 1 and Figure 2). When considered together with our previous physiological and metabolomic studies, as well as our studies of DNA methylation-related genes, these data suggest that the response of this medicinal plant to Cd stress is regulated at multiple levels rather than through a single defensive pathway [4,19]. In other words, the physiological damage and metabolic changes observed previously are likely part of a broader regulatory process that also involves transcriptome reprogramming and methylome changes. This is particularly important in *S. tonkinensis*, because Cd stress is relevant not only to plant growth and survival, but also to medicinal quality. The current results therefore provide a useful framework for linking earlier observations at the physiological and metabolic levels with molecular-level regulatory changes.

### 3.2. Transcriptional Reprogramming Under Cd Stress Suggests a Shift from Growth-Related Processes to Stress Adaptation

A striking feature of our data is the extent of transcriptional reprogramming under Cd stress, especially the much stronger repression observed under the higher Cd treatment (Figure 1A,B). This suggests that, as stress intensity increases, *S. tonkinensis* increasingly suppresses growth-related transcriptional programs while activating or maintaining pathways associated with stress adaptation. The recurrent enrichment of pathways related to photosynthesis, carbon metabolism, sulfur metabolism, and secondary metabolism across Cd treatments (Figure 1D) is consistent with this interpretation. Similar patterns have been reported in recent studies of Cd-stressed Kentucky bluegrass, rice, and Brassicaceae crops, in which adaptation was associated with broad changes in carbon allocation, ion balance, and membrane-related metabolism [31,32,33].

Our pathway analysis also highlighted transporter- and hormone-related pathways, particularly under stronger Cd stress (Figure 1D). These pathways are likely to be important for maintaining cellular homeostasis under metal toxicity. Sulfur metabolism is closely connected with cysteine and glutathione-related processes, which are central to detoxification, while ABC transporters may contribute to the transport and sequestration of Cd-related toxic compounds, chelates, or conjugates. The enrichment of hormone signaling pathways further suggests that Cd stress in *S. tonkinensis* involves active regulatory adjustment in addition to stress-induced injury. This is in agreement with recent studies showing that Cd can disrupt auxin-dependent growth regulation and that protective treatments affecting oxidative stress and photosynthetic injury can substantially alter downstream responses [34,35].

Taken together, these results suggest that Cd stress in *S. tonkinensis* leads to a reorganization of transcriptional priorities, with reduced emphasis on growth-related processes and greater involvement of detoxification, signaling, and metabolic adjustment.

### 3.3. CHH Methylation Appears to Be the Most Responsive Methylation Context Under Cd Stress

At the methylome level, CHH methylation showed the strongest response to Cd stress, whereas CG methylation remained the most highly methylated context overall (Figure 2B,C). These results indicate that the three sequence contexts respond differently to Cd stress. In our dataset, CG methylation showed comparatively limited variation, whereas CHH methylation was more responsive to stress conditions. The observation that DMRs were more frequently detected in promoter and flanking regions (Figure 3C,D) suggests that Cd stress-related methylation variation was often associated with gene-associated regulatory regions, rather than reflecting a broad, uniform rearrangement of methylation patterns across the genome. This interpretation is consistent with previous studies reporting stress-associated methylation dynamics in plants, particularly the high plasticity of non-CG methylation under stress conditions [36,37,38].

Genes showing hypermethylation accompanied by reduced expression were substantially more numerous than genes showing the opposite pattern, especially under the higher Cd treatment (Figure 5A,B). We focused on genes with inverse relationships between DNA methylation and transcript abundance because such patterns are commonly used as initial clues for identifying candidate loci potentially involved in methylation-associated transcriptional regulation, although they do not establish direct mechanistic causality by themselves [39,40]. Instead, they define a focused set of candidate genes whose methylation changes are associated with transcriptional responsiveness under Cd stress. The enrichment of these genes in photosynthesis-, lipid metabolism-, sulfur metabolism-, and secondary metabolism-related pathways (Figure 5C) suggests that methylation-associated expression changes are concentrated in biological processes likely to contribute to Cd adaptation.

### 3.4. The Present Results Link Cd Stress Responses to Medicinally Relevant Metabolic Regulation

A notable feature of this study is the recurrent identification of genes related to biosynthesis of secondary metabolites and isoquinoline alkaloid biosynthesis in the integrated methylome-transcriptome analysis (Figure 4C,D and Figure 5C). Similar Cd-associated changes in isoquinoline alkaloid biosynthesis have also been reported in another medicinal plant, *Coptis chinensis* [41]. In a medicinal plant such as *S. tonkinensis*, this finding is particularly relevant because Cd stress may affect not only conserved stress-responsive pathways but also secondary metabolic processes potentially related to medicinal quality, and DNA methylation has been increasingly recognized as an important regulatory layer in medicinal plants [42]. This interpretation is consistent with our previous study showing that Cd exposure altered the metabolite profile of *S. tonkinensis* [4]. Although photosynthesis, carbon metabolism, lipid metabolism, and sulfur metabolism represent conserved Cd-responsive processes, the methylation-associated changes in secondary metabolism and isoquinoline alkaloid biosynthesis provide a species-relevant perspective for this medicinal plant.

These findings also fit with our previous work on methylation-related genes in *S. tonkinensis*, in which Cd- and drought-responsive methylation machinery genes were identified [19]. In this sense, the current study extends earlier gene-family-level observations to the genome scale and suggests that Cd-induced metabolic responses may be associated with broader methylome plasticity. However, the present data do not directly demonstrate methylation-mediated regulation of specific active compounds. Further studies integrating methylome, transcriptome, and targeted metabolite profiling will be needed to clarify how DNA methylation changes may be linked to medicinally relevant metabolites under Cd stress.

### 3.5. Functional Validation of StGH3.1 Supports the Biological Relevance of Methylation-Associated Candidate Genes

An important extension of this study is the phenotype-based validation of *StGH3.1*, a hypomethylated/upregulated candidate gene identified by the integrated methylome–transcriptome analysis. Among the two shared hypomethylated/upregulated genes, *StGH3.1* was mapped to the plant hormone signal transduction pathway and annotated as a GH3 auxin-responsive gene, suggesting a potential link between methylation-associated transcriptional changes, hormone-related regulation, and Cd stress adaptation. Heterologous overexpression of *StGH3.1* in *N. benthamiana* improved growth under 100 and 200 μM CdCl_2_ treatment and was accompanied by increased SOD, POD, and CAT activities and reduced MDA accumulation (Figure 6). These results indicate that *StGH3.1* is functionally associated with Cd tolerance in the heterologous tobacco system.

Nevertheless, this validation should be interpreted cautiously. The present data do not establish a direct causal relationship between reduced methylation at the *StGH3.1* locus and its transcriptional activation. In addition, native knockout, knockdown, or complementation analyses in *S. tonkinensis* were not performed because a stable transformation system for this woody medicinal plant is still under development. Hormone profiling, including IAA and JA-Ile quantification, was also not conducted. Therefore, the native function of *StGH3.1* in *S. tonkinensis* and its potential link with hormone metabolism require further validation.

## 4. Materials and Methods

### 4.1. Plant Materials and Cd Treatments

The Cd treatment system followed our previously published physiological and metabolomic study of *S. tonkinensis* [4]. Briefly, seedlings were treated with 0, 20, 40, 60, and 80 μM CdCl_2_, designated as T0, T1, T2, T3, and T4, respectively. Based on the previously reported phenotypic, physiological, and metabolomic responses, T0, T2, and T4 were selected for the present RNA-seq and WGBS analyses to represent the control, moderate Cd stress, and severe Cd stress conditions, respectively. T1 caused no obvious phenotypic changes, whereas T2 induced visible leaf curling and clear physiological responses, and T4 caused the strongest growth inhibition, oxidative damage, and Cd accumulation. A summary of the treatment design and selection rationale is provided in Supplementary Table S3.

For both RNA-seq and WGBS analyses, each treatment group included three independent biological replicates, and each biological replicate consisted of pooled root tissues from six individual plants. After 10 days of treatment, root samples were harvested, thoroughly rinsed with ultrapure water to remove residual surface ions, immediately frozen in liquid nitrogen, and stored at −80 °C until RNA and DNA extraction.

### 4.2. RNA Extraction, Library Construction, and Transcriptome Sequencing

Total RNA was extracted from frozen root tissues using TRIzol reagent (Invitrogen, Carlsbad, CA, USA). RNA quality and integrity were assessed using an Agilent 2100 Bioanalyzer and RNase-free agarose gel electrophoresis. After total RNA extraction, mRNA was enriched using Oligo(dT) beads, fragmented into short fragments, and reverse-transcribed into cDNA using the NEBNext Ultra RNA Library Prep Kit for Illumina (NEB #7530, New England Biolabs, Ipswich, MA, USA). The purified double-stranded cDNA fragments were end-repaired, A-tailed, ligated to Illumina sequencing adapters, size-selected, PCR-amplified, and sequenced on the Illumina NovaSeq X Plus platform (Illumina Inc., San Diego, CA, USA) by Gene Denovo Biotechnology Co., Ltd. (Guangzhou, China).

Raw reads were filtered using fastp v0.18.0 [43] by removing reads containing adapters, reads containing more than 10% unknown nucleotides, and low-quality reads containing more than 50% bases with Q ≤ 20. rRNA reads were removed by mapping to the rRNA database using Bowtie2 v2.2.8 [44]. The remaining clean reads were mapped to the reference genome using HISAT2 v2.4 [45]. Mapped reads were assembled using StringTie v1.3.1, and gene expression abundance was estimated as FPKM values using RSEM v1.3.1 [46,47,48]. Differential expression analysis was performed using DESeq2 v1.30.0 based on raw read counts. Default independent filtering was applied, and log2 fold-change shrinkage was performed before downstream DEG filtering. Genes with FDR < 0.05 and |log2 fold change| ≥ 1 were considered differentially expressed genes (DEGs) [49]. GO and KEGG enrichment analyses were performed using a hypergeometric test, and terms/pathways with FDR ≤ 0.05 were regarded as significantly enriched.

### 4.3. Genomic DNA Extraction, WGBS Library Construction, and Methylation Calling

Genomic DNA was extracted from the same root samples used for transcriptome analysis. DNA concentration and integrity were assessed using a NanoPhotometer spectrophotometer and agarose gel electrophoresis, respectively. For WGBS, genomic DNA was fragmented into 100–300 bp fragments by sonication, purified, end-repaired, A-tailed, ligated to methylated sequencing adapters, bisulfite-converted using the EZ DNA Methylation-Gold Kit (Zymo Research, Irvine, CA, USA), PCR-amplified, and sequenced on the Illumina HiSeq 2500 platform by Gene Denovo Biotechnology Co., Ltd., Guangzhou, China.

Raw bisulfite reads were filtered by removing reads containing more than 10% unknown nucleotides and low-quality reads containing more than 40% bases with Q ≤ 20. Clean reads were mapped to the previously assembled *S. tonkinensis* reference genome (GSA, PRJCA053979) using BSMAP v2.90 [50]. Methylated cytosines were identified using a custom Perl script after correction for bisulfite non-conversion rate according to Lister et al. [51]. The per-sample WGBS sequencing statistics, including mapping rate and average sequencing depth, are provided in Supplementary Table S2.

### 4.4. Identification and Annotation of DMRs and DMGs

Differentially methylated cytosines (DMCs) and differentially methylated regions (DMRs) between Cd-treated and control groups were identified for each methylation context using methylKit v1.7.10 [52]. Differential DNA methylation at each cytosine locus was tested using Pearson’s chi-square test. The minimum read coverage required to call the methylation status of a cytosine was set to 4. DMCs were identified separately for each sequence context using q ≤ 0.05 and context-specific methylation-difference thresholds: |methylation difference| ≥ 0.25 for CG, ≥0.25 for CHG, ≥0.15 for CHH, and ≥0.20 for all C contexts.

DMRs were identified using the same minimum cytosine coverage threshold and context-specific criteria. For CG and CHG contexts, regions were retained as DMRs when each window contained at least 5 cytosines, with |methylation difference| ≥ 0.25 and q ≤ 0.05. For CHH contexts, each window was required to contain at least 15 cytosines, with |methylation difference| ≥ 0.15 and q ≤ 0.05. For all C contexts, each window was required to contain at least 20 cytosines, with |methylation difference| ≥ 0.20 and q ≤ 0.05. DMRs were further annotated to genomic features, including promoter, exon, intron, gene body, upstream, and downstream regions. Promoter regions were defined as the 2-kb regions upstream of transcription start sites. For methylation profile and distribution analyses, flanking regions referred to the 2-kb regions upstream and downstream of annotated genes. Genes whose promoter, gene body, or flanking regions overlapped with DMRs were defined as differentially methylated genes (DMGs).

### 4.5. Integrated Analysis of Methylome and Transcriptome Datasets

DEGs and DMGs were integrated based on gene identifiers to identify candidate methylation-associated transcriptional changes. Genes showing simultaneous changes in DNA methylation and transcript abundance were classified according to the direction of change, with particular attention to inverse methylation-expression patterns, including hypermethylated/downregulated and hypomethylated/upregulated groups. Genes showing inverse changes in DNA methylation and gene expression were selected as candidate methylation-associated regulatory genes for downstream analysis. Shared gene sets were visualized using Venn diagrams. GO enrichment analysis was performed against the whole-genome background using a hypergeometric test, and KEGG pathway enrichment or annotation analysis was conducted for the integrated gene sets.

### 4.6. Functional Validation of StGH3.1 in Transgenic N. benthamiana Under Cd Stress

The coding sequence of *StGH3.1* was amplified from *S. tonkinensis* cDNA and inserted into the plant expression vector pBK-35S-GLosGFP. The recombinant construct was introduced into *Agrobacterium tumefaciens* strain EHA105 and used for *N. benthamiana* transformation through Agrobacterium-mediated leaf-disc transformation. PCR-positive transgenic *N. benthamiana* plants were screened on Basta-containing medium, and qRT-PCR analysis was performed using cDNA reverse-transcribed from total RNA extracted from independent transgenic lines, with relative *StGH3.1* expression levels calculated by the 2^−ΔΔCt^ method using *N. benthamiana Actin* as the internal reference.

Wild-type and transgenic *N. benthamiana* seedlings were cultured under control conditions or treated with 100 μM and 200 μM CdCl_2_ for 30 d. Plant growth phenotypes were recorded after treatment. Leaf samples were then collected for the determination of POD, CAT, and SOD activities and MDA content using routine biochemical assays. Physiological measurements were compared between the transgenic lines and the wild type under the same treatment conditions.

## 5. Conclusions

This study demonstrates that Cd stress in *S. tonkinensis* is accompanied by coordinated changes in DNA methylation and gene expression. CHH methylation was the most responsive context, and Cd-induced DMRs were more frequently detected in promoter and flanking regions, suggesting that methylation variation at gene-associated regions may be linked to transcriptional responses under Cd stress. Integrated methylome–transcriptome analysis identified candidate genes with concurrent methylation and expression changes, including genes associated with photosynthesis, lipid metabolism, sulfur-related metabolism, secondary metabolic pathways potentially related to medicinal quality, and hormone-related regulation. Among the shared hypomethylated/upregulated genes, *StGH3.1* was mapped to the plant hormone signal transduction pathway and was selected for heterologous validation. Overexpression of *StGH3.1* in *N. benthamiana* enhanced Cd tolerance, as shown by improved growth, increased antioxidant enzyme activities, and reduced MDA accumulation under Cd stress. Together, these findings provide genome-wide evidence that DNA methylation is associated with transcriptional reprogramming during Cd stress and identify *StGH3.1* as a methylation-associated candidate gene whose heterologous overexpression enhances Cd tolerance in *N. benthamiana*.

## Data Availability

The raw sequencing data from this study have been deposited in the Genome Sequence Archive in BIG Data Center (https://bigd.big.ac.cn/), Beijing Institute of Genomics (BIG), Chinese Academy of Sciences, under the accession number: CRA043133.

## Supplementary Materials

The following supporting information can be downloaded at: https://www.mdpi.com/article/10.3390/plants15121861/s1, Table S1. Summary of transcriptome sequencing data of the samples. Table S2. Summary of WGBS data of the samples. Table S3. Cd treatment design and rationale for selecting T0, T2, and T4 for RNA-seq and WGBS analyses. Figure S1. Relationships between promoter DNA methylation and gene expression in *S. tonkinensis* under Cd stress. Boxplots show the distribution of gene expression levels [log2(FPKM + 1)] for genes grouped by promoter methylation levels in the CG, CHG, and CHH contexts under T0, T2, and T4 treatments. Figure S2. Relationships between gene body DNA methylation and gene expression in *S. tonkinensis* under Cd stress. Boxplots show the distribution of gene expression levels [log2(FPKM + 1)] for genes grouped by gene body methylation levels in the CG, CHG, and CHH contexts under T0, T2, and T4 treatments. Figure S3. Locus-level DNA methylation profile of *StGH3.1* under Cd stress. The gene structure and WGBS methylation profiles of *StGH3.1* in T0, T2, and T4 are shown. Blue boxes indicate exons, and the arrow direction indicates transcription orientation. The yellow highlighted region indicates the differentially methylated region (DMR), which is located in the upstream region of the gene. Figure S4. qRT-PCR analysis of *StGH3.1* expression in independent transgenic *N. benthamiana* lines.
