# Supplementary material for: Integrated Methylome and Transcriptome Analyses Reveal Methylation-Associated Cadmium Stress Responses in Sophora tonkinensis
Source: Plants (Basel). 2026 Jun 16;15(12):1861. doi: 10.3390/plants15121861 (PMC13306473; doi:10.3390/plants15121861)
Supplement: Supplementary file 1 [file plants-15-01861-s001.zip › Supplementary Figure.pdf]

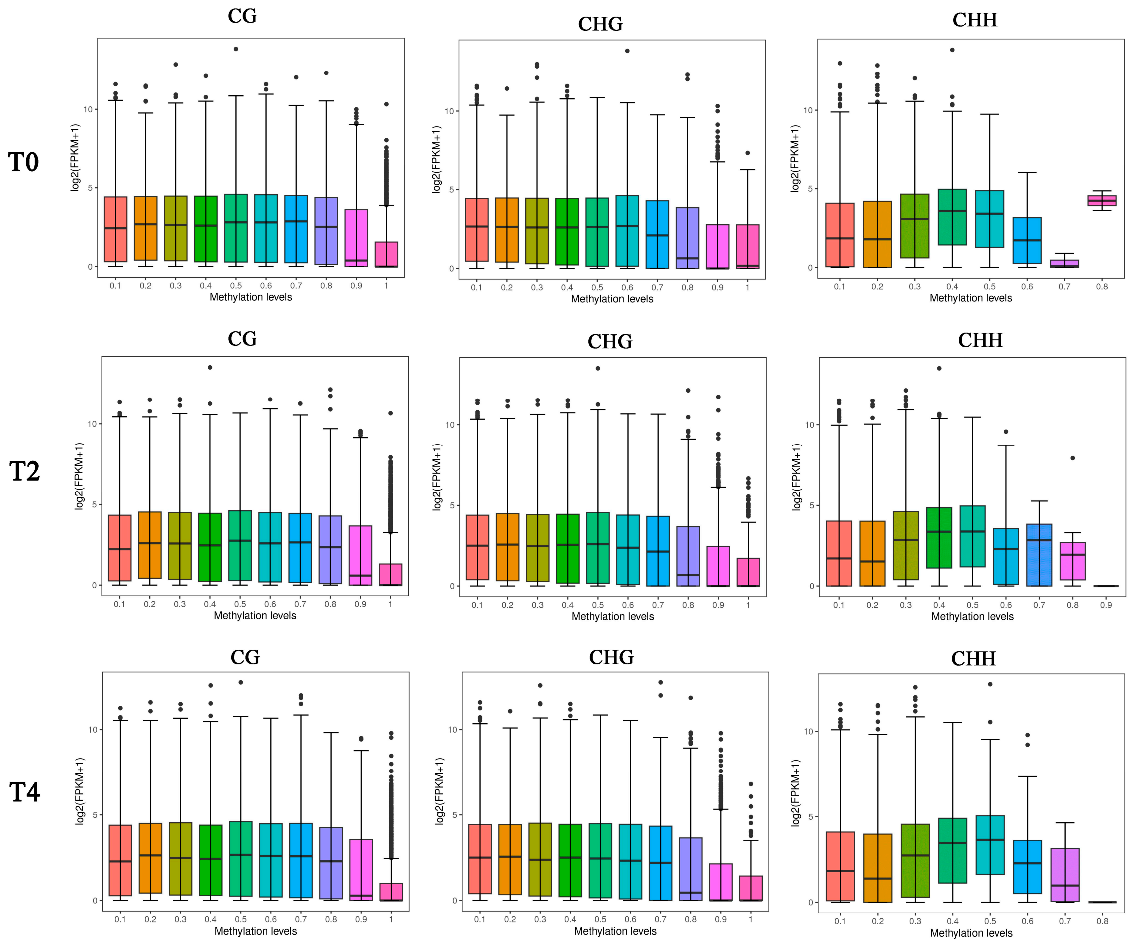

Figure S1. Relationships between promoter DNA methylation and gene expression in *S. tonkinensis* under Cd stress. Boxplots show the distribution of gene expression levels [log2(FPKM + 1)] for genes grouped by promoter methylation levels in the CG, CHG, and CHH contexts under T0, T2, and T4 treatments.

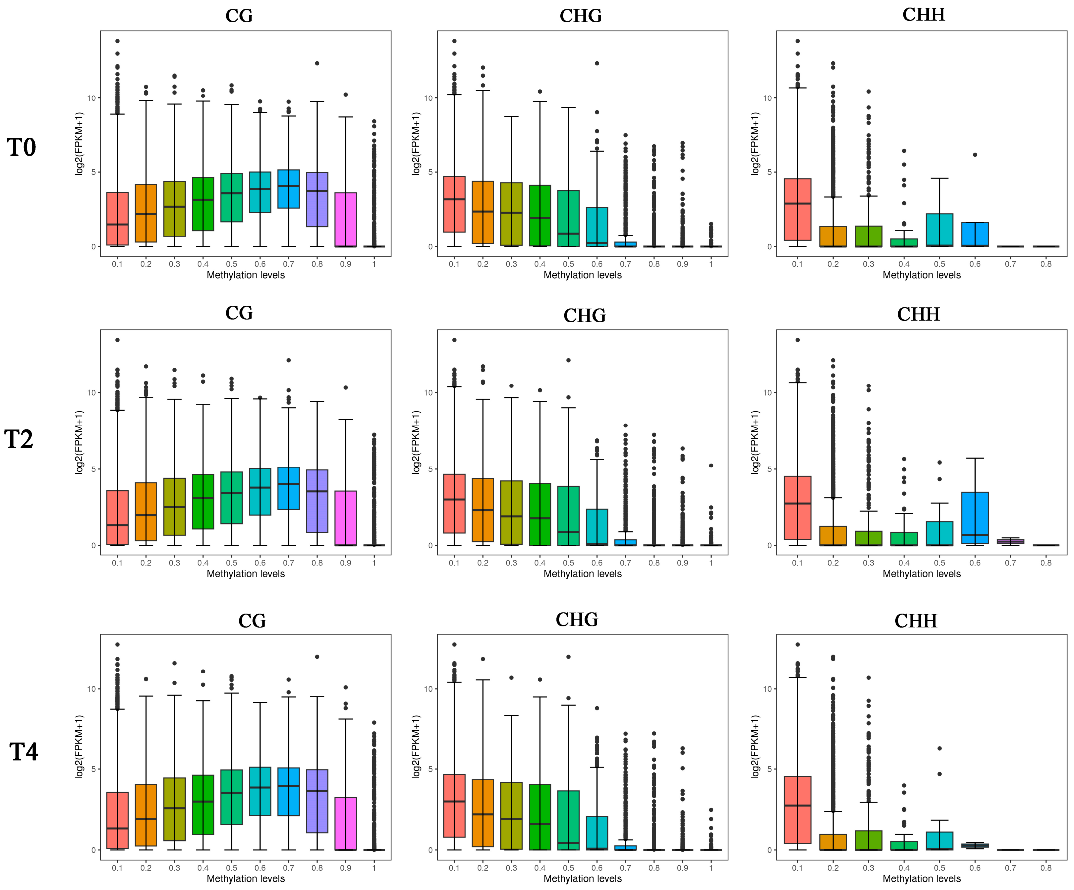

Figure S2. Relationships between gene body DNA methylation and gene expression in *S. tonkinensis* under Cd stress. Boxplots show the distribution of gene expression levels [log2(FPKM + 1)] for genes grouped by gene body methylation levels in the CG, CHG, and CHH contexts under T0, T2, and T4 treatments.

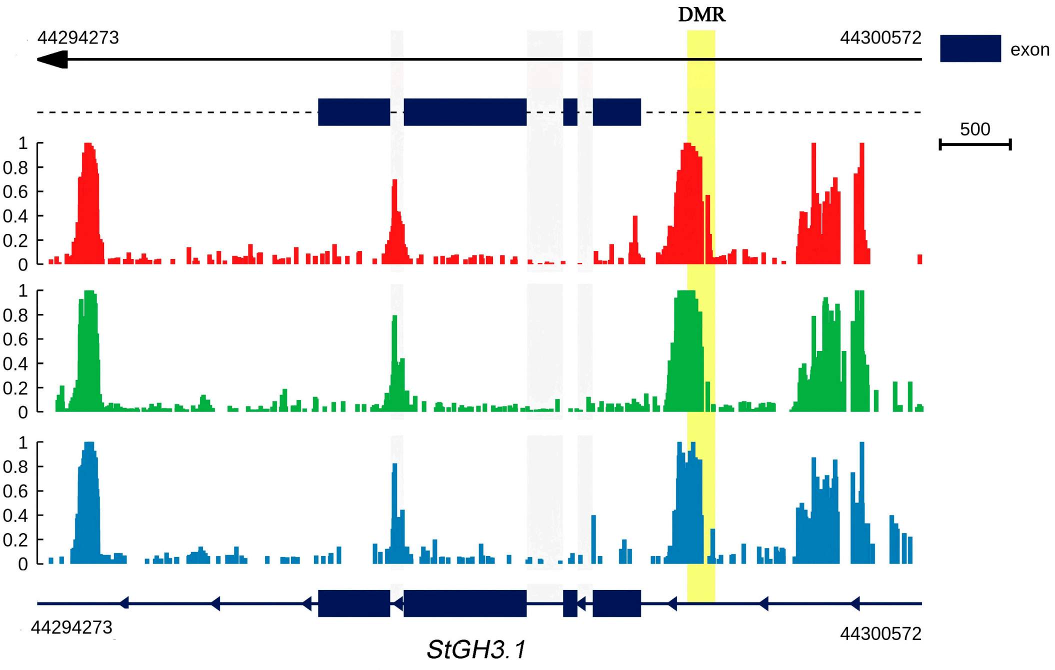

Figure S3. Locus-level DNA methylation profile of *StGH3.1* under Cd stress. The gene structure and WGBS methylation profiles of *StGH3.1* in T0, T2, and T4 are shown. Blue boxes indicate exons, and the arrow direction indicates transcription orientation. The yellow highlighted region indicates the differentially methylated region (DMR), which is located in the upstream region of the gene.

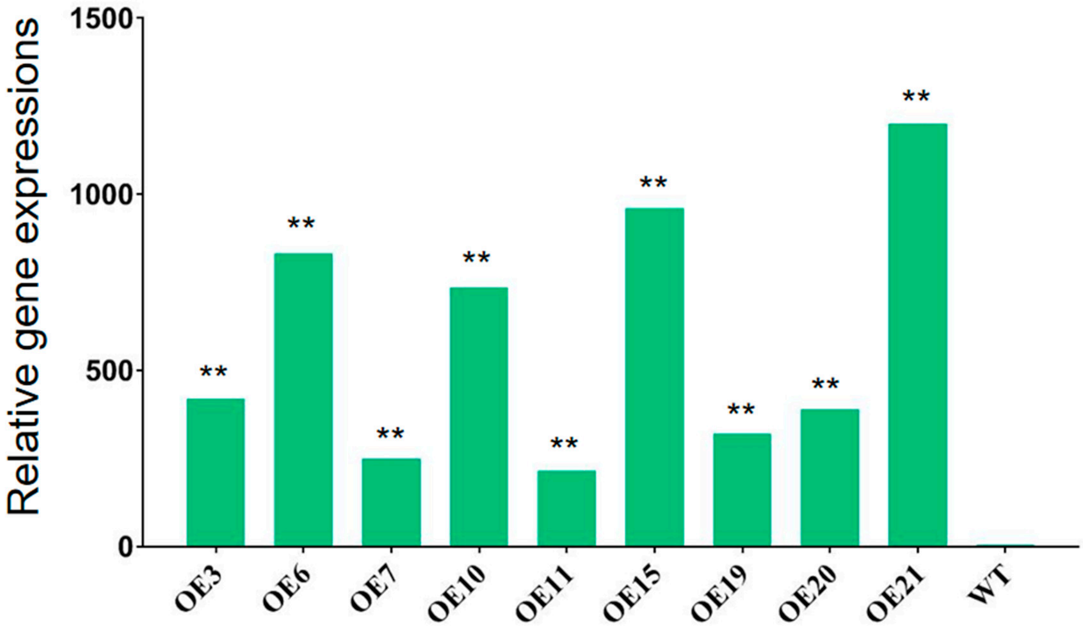

Figure S4. qRT-PCR analysis of *StGH3.1* expression in independent transgenic *N. benthamiana* lines.
